# Supplementary material for: Intermolecular interactions play a role in the distribution and transport of charged contrast agents in a cartilage model
Source: PLoS One. 2019 Oct 3;14(10):e0215047. doi: 10.1371/journal.pone.0215047 (PMC6776344; doi:10.1371/journal.pone.0215047)
Supplement: S1 Appendix — (PDF) [file pone.0215047.s001.pdf]

S1 Appendix. Diffusion-driven transport of ions in an infinite, one-dimensional Donnan system

The system depicted in Figure 1A is considered, with the exception that we treat any ion, X, with significantly lower concentration than that of the Na^+ and Cl^- ions. First, the equations that describe the concentration step in Figure 4A as well as the concentration profile in the insert of Figure 4A will be presented. Subsequently, the equations describing the concentration profile in Figure 4A will be given.

The transport of an ionic species i is given by (in one dimension):

$$J_i(z) = -D_i(z)\frac{\partial c_i(z)}{\partial z} - \frac{D_i(z)c_i(z)}{RT}\frac{\partial \mu_i^{\mathrm{corr}}(z)}{\partial z} - \frac{Fz_iD_i(z)c_i(z)}{RT}\frac{\partial \phi(z)}{\partial z} \tag{A}$$

where J_i is the diffusive flux of ion species i, D_i and c_i are the diffusion coefficient and the concentration of i, respectively, z is the direction, R is the gas constant and T is the temperature. μ_i^{corr} is the corrected chemical potential of species i (resulting from non-ideal intermolecular interactions). Furthermore, z_i is the charge of species i, F is the Faraday constant and ϕ is the electric potential. There is a local quasi steady-state around $z = 0$ that gives rise to the discontinuity in concentration and potential across the interface between the salt and polyelectrolyte solutions. From Eq A this means that J_i can be set constant, and since the gradients in concentration and electric potential in the local region around $z = 0$ are much larger than the macroscopic changes, the flux can also be set to zero in the interval $0^- \leq z \leq 0^+$:

$$(0^- \leq z \leq 0^+) \qquad c_i(z) = C^i_{\mathrm{s}(0^+)}\exp\left(-\frac{\left(\mu_i^{\mathrm{corr}}(z)-\mu_i^{\mathrm{corr}}(0^+)\right)}{RT} - \frac{z_iF\left(\phi(z)-\phi(0^+)\right)}{RT}\right) \tag{B}$$

where $C^i_{\mathrm{s}(0^+)}$ is the concentration of ion i at position 0^+, $\mu_i^{\mathrm{corr}}(0^+)$ is the corrected chemical potential of ion i at position 0^+ and $\phi(0^+)$ is the electric potential at position 0^+. If the following holds:

$$\left|\frac{\left(\mu_i^{\mathrm{corr}}(z)-\mu_i^{\mathrm{corr}}(0^+)\right)}{RT} + \frac{z_iF\left(\phi(z)-\phi(0^+)\right)}{RT}\right| < 1 \tag{C}$$

then a Taylor expansion of the exponential term in Eq B yields:

$$(0^- \leq z \leq 0^+) \qquad c_i(z) \approx C^i_{\mathrm{s}(0^+)}\left(1-\left(\frac{\left(\mu_i^{\mathrm{corr}}(z)-\mu_i^{\mathrm{corr}}(0^+)\right)}{RT} + \frac{z_iF\left(\phi(z)-\phi(0^+)\right)}{RT}\right)\right) \tag{D}$$

Given the assumptions stated above, the step size in the concentration indicated in Figure 4A ($\Delta c_i \approx C^i_{\mathrm{p}(0^-)} - C^i_{\mathrm{s}(0^+)}$), is the macroscopic step between the two solutions. It is obtained from:

$$\Delta c_i = -C^i_{\mathrm{s}(0^+)}\left(\frac{\Delta\mu_i^{\mathrm{corr}}}{RT} + \frac{z_iF}{RT}\Delta\phi\right) \tag{E}$$

where $\Delta\mu_i^{\mathrm{corr}} = \mu_{i,\mathrm{p}}^{\mathrm{corr}} - \mu_{i,\mathrm{s}}^{\mathrm{corr}}$, which means we assume that the correction factor for the chemical potential is independent of position in the salt and polyelectrolyte solutions, respectively. $\Delta\phi = \phi(0^-)-\phi(0^+)$ is the electric potential difference between the polyelectrolyte and salt solution on a macroscopic scale. $\Delta\phi$ is obtained from Poisson's equation:

$$\epsilon_0\epsilon_\mathrm{r}\frac{\partial^2\phi}{\partial z^2} = -F\sum_i z_ic_i - Fz_{\mathrm{p}^-}c_{\mathrm{p}^-,\mathrm{p}} \tag{F}$$

where ϵ_0 is the permittivity of vacuum, ϵ_r, is the dielectric constant, z_{p^-} is the valency of the polyelectrolyte and $c_{\mathrm{p}^-,\mathrm{p}}$ is the concentration of the polyelectrolyte. If Eq D is inserted into Poisson's equation, the following expressions are obtained:

$$(0^- \leq z < 0) \qquad \epsilon_0\epsilon_\mathrm{r}\frac{\partial^2\phi}{\partial z^2} = \left(\phi(z)-\phi(0^+)\right)\frac{F^2}{RT}\sum_i z_i^2C^i_{\mathrm{s}(0^+)} + \frac{F}{RT}\sum_i z_iC^i_{\mathrm{s}(0^+)}\Delta\mu_i^{\mathrm{corr}} - Fz_{\mathrm{p}^-}c_{\mathrm{p}^-,\mathrm{p}} \tag{G}$$

$$(0 < z \leq 0^+) \qquad \epsilon_0\epsilon_\mathrm{r}\frac{\partial^2\phi}{\partial z^2} = \left(\phi(z)-\phi(0^+)\right)\frac{F^2}{RT}\sum_i z_i^2C^i_{\mathrm{s}(0^+)} \tag{H}$$

If ϵ_r is assumed to be the same in the polyelectrolyte solution and in the salt solution, and by using the boundary condition that the potential and the gradient of the potential should be continuous at $z = 0$, the following solutions of these differential equations are obtained:

$$(0^- \le z < 0) \qquad \phi(z) - \phi(0^+) = \Delta\phi\left(1 - \frac{e^{\kappa z}}{2}\right) \tag{I}$$

$$(0 < z \le 0^+) \qquad \phi(z) - \phi(0^+) = \frac{\Delta\phi}{2} e^{-\kappa z} \tag{J}$$

where

$$\kappa = \sqrt{\frac{F^2 \sum_i z_i^2 C^i_{\mathrm{s}(0^+)}}{\epsilon_0 \epsilon_r RT}} = \tag{K}$$

and

$$\Delta\phi = \frac{RT}{F \sum_i z_i^2 C^i_{\mathrm{s}(0^+)}} \left(z_{\mathrm{p}^-} c_{\mathrm{p}^-,\mathrm{p}} - \sum_i z_i C^i_{\mathrm{s}(0^+)} \frac{\Delta\mu_i^{\mathrm{corr}}}{RT} \right) \tag{L}$$

We next turn to the concentration profile of ion X over a macroscopic length scale and over time. The change of ion concentration over time, t, is described by the following equation:

$$\frac{\partial c_X}{\partial t} = -\frac{\partial}{\partial z}\left(-D_X(z)\frac{\partial c_X(z)}{\partial z} - \frac{D_X(z)c_X(z)}{RT}\frac{\partial \mu_X^{\mathrm{corr}}(z)}{\partial z} - \frac{F z_X D_X(z) c_X(z)}{RT}\frac{\partial \phi(z)}{\partial z} \right) \tag{M}$$

By solving this equation, the concentration of species X in the system at different times can be obtained, as in Figure 2. To find an analytic for each solution, some further assumptions have to be made.

- The polyelectrolyte and salt solutions are assumed to be infinite in the negative and positive z-direction, respectively.
- μ_i^{corr} is assumed to have a constant value in each compartment, which is a reasonable assumption, since we are dealing with small concentration differences over time. Furthermore, previous Monte Carlo simulations of $Gd(DTPA)^{2-}$ showed that at low concentrations, the $Gd(DTPA)^{2-}$ - $Gd(DTPA)^{2-}$ interactions do not have to be taken into account when the excess chemical potential is obtained (Algotsson 2012). We assume that these conclusions can be extended to any specie X present in low concentration.
- The concentration of ion X is significantly lower than the concentration of Na^+ and Cl^- ions. Therefore, the influence from X on the electric potential is negligible, making it possible to neglect the last term in Eq M.

With these assumptions and excluding the region $0^- < z < 0^+$, Eq M reduces to the following equation:

$$\frac{\partial c_X}{\partial t} = D_X \frac{\partial^2 c_X}{\partial z^2} \tag{N}$$

with the conditions:

$$c_X(z = 0^-, t) = C^X_{\mathrm{p}(0^-)} \tag{O}$$

$$c_X(z = 0^+, t) = C^X_{\mathrm{s}(0^+)} \tag{P}$$

Furthermore, the following initial conditions are assumed:

$$c_X(z, 0) = \begin{cases} 0 & z < 0 \\ C^X_{\mathrm{s},0} & z > 0 \end{cases} \tag{Q}$$

Eq N can then be solved for the X ion:

$$(z \le 0^-) \qquad c_{X,\mathrm{p}}(z,t) = C^X_{\mathrm{p}(0^-)}\left(1 + \mathrm{erf}\left(\frac{z}{\sqrt{4D_{X,\mathrm{p}}t}}\right)\right) \tag{R}$$

$$(z \ge 0^+) \qquad c_{X,\mathrm{s}}(z,t) = C^X_{\mathrm{s}(0^+)} + \left(C^X_{\mathrm{s},0} - C^X_{\mathrm{s}(0^+)}\right)\mathrm{erf}\left(\frac{z}{\sqrt{4D_{X,\mathrm{s}}t}}\right) \tag{S}$$

where $D_{X,\mathrm{p}}$ and $D_{X,\mathrm{s}}$ are the diffusion coefficients of ion X in the polyelectrolyte solution and the salt solution, respectively.
Furthermore, from the condition that the flux of ions is equal on both sides of $z = 0$, the following equation is obtained:

$$C^X_{\mathrm{s}(0^+)} = \frac{C^X_{s,0}}{1 + \alpha\sqrt{D_{X,\mathrm{p}}/D_{X,\mathrm{s}}}} \tag{T}$$

where

$$\alpha = \frac{C^X_{\mathrm{p}(0^-)}}{C^X_{\mathrm{s}(0^+)}} \tag{U}$$

can be obtained from Eq E.

Influence of a membrane between the polyelectrolyte and salt solutions

A membrane between the polyelectrolyte solution and the salt solution will affect the flux of ions over the interface. We assume that there is a membrane between $0 < z < L_\mathrm{m}$ and that the diffusion coefficient of ion X in the membrane is $D_{X,\mathrm{m}}$. For short times, the concentration in the membrane is described by Eqs R, S and T, with $D_{X,\mathrm{s}}$ replaced by $D_{X,\mathrm{m}}$. This is approximately valid as long as:

$$t < L_\mathrm{m}^2/4D_{X,\mathrm{m}} \tag{V}$$

For intermediate times, it is a non-trivial task to determine the concentrations in the different compartments in the system. However, at longer times, the solution will approach Eqs R and S (with Eq S valid in the interval $z > L_\mathrm{m}$), while the following equation approximately describes the concentration in the membrane:

$$(0^+ < z < L_\mathrm{m}) \qquad c_X(z,t) = c_{\mathrm{Eq\ L}}(L_\mathrm{m}, t) + (z - L_\mathrm{m})\frac{\Delta c_{X,\mathrm{m}}}{\Delta L_\mathrm{m}} \tag{W}$$

where

$$\frac{\Delta c_{X,\mathrm{m}}}{\Delta L_\mathrm{m}} = \frac{\alpha C^X_{\mathrm{s}(0^+)}}{D_{X,\mathrm{m}}}\sqrt{\frac{D_{X,\mathrm{p}}}{\pi t}} = \frac{\alpha}{D_{X,\mathrm{m}}}\sqrt{\frac{D_{X,\mathrm{p}}}{\pi t}}\frac{C^X_{\mathrm{s},0}}{1 + \alpha\sqrt{D_{X,\mathrm{p}}/D_{X,\mathrm{s}}}} \tag{X}$$

The concentration profiles, including effects of convection in the salt solution

When determining the diffusion coefficient in the polyelectrolyte solution, the existence of temperature-driven convection in the salt solution has to be considered. In the current experiments, the influence of convection decreased with time and was neglected at longer times. Therefore, the concentration profiles derived in the previous section with no convection have to be modified. We consider a time interval $t > t_\mathrm{start}$, where it is experimentally observed that $C^X_{\mathrm{p}(0^-)}$ is constant. It was found that for the current data, the concentration in the polyelectrolyte solution at the start of the time interval, t_start, could be fitted to:

$$(z \le 0^-) \qquad c_{X,\mathrm{p}}(z, t_\mathrm{start}) = C^X_{\mathrm{p}(0^-)}\left(1 + \mathrm{erf}\left(\frac{z}{w}\right)\right) \tag{Y}$$

where w is an empirical parameter characterizing the length scale over which the concentration profile in the polyelectrolyte solution changes. Since convection in the polyelectrolyte solution is assumed to be negligible, the following solution to the concentration in the polyelectrolyte is obtained at times $t > t_\mathrm{start}$:

$$(z \le 0^-) \qquad c_{X,\mathrm{p}}(z,t) = C^X_{\mathrm{p}(0^-)}\left(1 + \mathrm{erf}\left(\frac{z}{\sqrt{4D_{X,\mathrm{p}}(t - t_\mathrm{start}) + w^2}}\right)\right) \tag{Z}$$

The parameter w is obtained by fitting Eq Y to the experimental concentration profile at time t_start. Subsequently, $D_{X,\mathrm{p}}$ can be determined by fitting Eq Z to the concentration profiles at time $t > t_\mathrm{start}$ (see Figure A for an example). It should also be noted that these expressions, and the procedure to determine the diffusion coefficient, are valid only at time points where $t < t_\mathrm{boundary}$, where t_boundary is the time it takes for the ionic species X to diffuse to the boundary of the container (approximately on the order of 10 h in the current experiments).

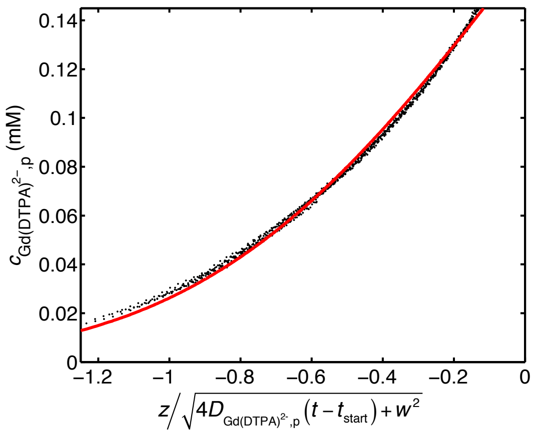

FIG. A: Experimental data (points) from different times and positions of the concentration of $Gd(DTPA)^{2-}$ in the polyelectrolyte solution with a FCD value of -108 mM fitted to a modified expression of Eq 18 (Eq Z) (solid line) yielding $D_{Gd(DTPA)^{2-},p}$.
