# Supplementary material for: Intermolecular interactions play a role in the distribution and transport of charged contrast agents in a cartilage model
Source: PLoS One. 2019 Oct 3;14(10):e0215047. doi: 10.1371/journal.pone.0215047 (PMC6776344; doi:10.1371/journal.pone.0215047)
Supplement: S2 Appendix — (PDF) [file pone.0215047.s002.pdf]

S2 Appendix. Donnan equilibrium

At equilibrium, the chemical potential for ion species i is the same in the salt solution as in the polyelectrolyte solution ($\mu_{i,\mathrm{s}} = \mu_{i,\mathrm{p}}$). Since the electric potential difference between the two solutions depends on all ions and not only on species i alone, the following is obtained from Equation B in S1 Appendix for the case studied here:

$$\frac{C^{\mathrm{Na^+}}_{\mathrm{p}(0^-)}}{C^{\mathrm{Na^+}}_{\mathrm{s}(0^+)}} \exp\left(\frac{\Delta\mu^{\mathrm{corr}}_{\mathrm{Na^+}}}{RT}\right) = \frac{C^{\mathrm{Cl^-}}_{\mathrm{s}(0^+)}}{C^{\mathrm{Cl^-}}_{\mathrm{p}(0^-)}} \exp\left(-\frac{\Delta\mu^{\mathrm{corr}}_{\mathrm{Cl^-}}}{RT}\right) = \sqrt{\frac{C^{\mathrm{G.^{2-}}}_{\mathrm{s}(0^+)}}{C^{\mathrm{G.^{2-}}}_{\mathrm{p}(0^-)}} \exp\left(-\frac{\Delta\mu^{\mathrm{corr}}_{\mathrm{G.^{2-}}}}{2RT}\right)} \tag{A}$$

Furthermore, due to the electro-neutrality conditions, and assuming that $c_{\mathrm{G.^{2-}}} \ll c_{\mathrm{Cl^-}}$, FCD $= z_{\mathrm{p^-}} c_{\mathrm{p^-,p}}$, the following holds in the salt solution:

$$c_{\mathrm{Na^+,s}} - c_{\mathrm{Cl^-,s}} = 0 \tag{B}$$

and in the polyelectrolyte solution:

$$c_{\mathrm{Na^+,p}} - c_{\mathrm{Cl^-,p}} + \mathrm{FCD} = 0 \tag{C}$$

Eqs A, B and C gives:

$$\mathrm{FCD} = \frac{c^2_{\mathrm{Na^+,s}}}{c_{\mathrm{Na^+,p}}} \exp\left(\frac{-\left(\Delta\mu^{\mathrm{corr}}_{\mathrm{Na^+}} + \Delta\mu^{\mathrm{corr}}_{\mathrm{Cl^-}}\right)}{RT}\right) - c_{\mathrm{Na^+,p}} \tag{D}$$

$$\mathrm{FCD} = c_{\mathrm{Cl^-,p}} - \frac{c^2_{\mathrm{Cl^-,s}}}{c_{\mathrm{Cl^-,p}}} \exp\left(\frac{-\left(\Delta\mu^{\mathrm{corr}}_{\mathrm{Na^+}} + \Delta\mu^{\mathrm{corr}}_{\mathrm{Cl^-}}\right)}{RT}\right) \tag{E}$$

$$\mathrm{FCD} = c_{\mathrm{Na^+},s}\left(\sqrt{\frac{c_{\mathrm{G.^{2-},p}}}{c_{\mathrm{G.^{2-},s}}}} \exp\left(\frac{-\left(\Delta\mu^{\mathrm{corr}}_{\mathrm{Cl^-}} - \frac{\Delta\mu^{\mathrm{corr}}_{\mathrm{G.^{2-}}}}{2}\right)}{RT}\right) - \sqrt{\frac{c_{\mathrm{G.^{2-},s}}}{c_{\mathrm{G.^{2-},p}}}} \exp\left(\frac{-\left(\Delta\mu^{\mathrm{corr}}_{\mathrm{Na^+}} + \frac{\Delta\mu^{\mathrm{corr}}_{\mathrm{G.^{2-}}}}{2}\right)}{RT}\right)\right) \tag{F}$$
