# Supplementary material for: Intermolecular interactions play a role in the distribution and transport of charged contrast agents in a cartilage model
Source: PLoS One. 2019 Oct 3;14(10):e0215047. doi: 10.1371/journal.pone.0215047 (PMC6776344; doi:10.1371/journal.pone.0215047)
Supplement: S3 Appendix — (PDF) [file pone.0215047.s003.pdf]

S3 Appendix. Determination of dialysis time

A control experiment was carried out in order to determine the time needed for the dialysis in order to reach equilibrium between the polyelectrolyte and salt solutions. Thus 7 mL 2.6 wt% CMC solution were dialyzed against 1.5 L 150 mM NaCl solution, where the latter was changed every day. The dialyses were performed in four corresponding setups and were stopped after different times, after which the sodium content in the solutions was determined by elementary analysis. The results are shown in Figure A where it can be seen that between day 1 and day 5 the sodium content is almost constant, which indicates that equilibrium is reached at this time. After 6 days, the sodium content is seen to decrease, probably on account of degradation of the polyelectrolyte. To minimize the degradation of the polyelectrolyte, but still reach equilibrium, a dialysis time of 1 day was chosen based on these results.

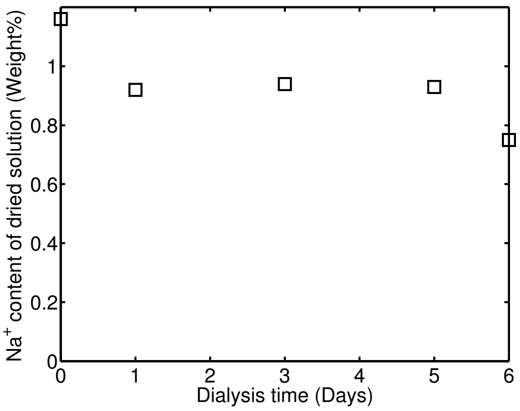

FIG. A: Control experiment used for the determination of the dialysis time.
