# Supplementary material for: Intermolecular interactions play a role in the distribution and transport of charged contrast agents in a cartilage model
Source: PLoS One. 2019 Oct 3;14(10):e0215047. doi: 10.1371/journal.pone.0215047 (PMC6776344; doi:10.1371/journal.pone.0215047)
Supplement: S1 Fig — (PDF) [file pone.0215047.s004.pdf]

S1 Figure. Additional experimental concentration profiles

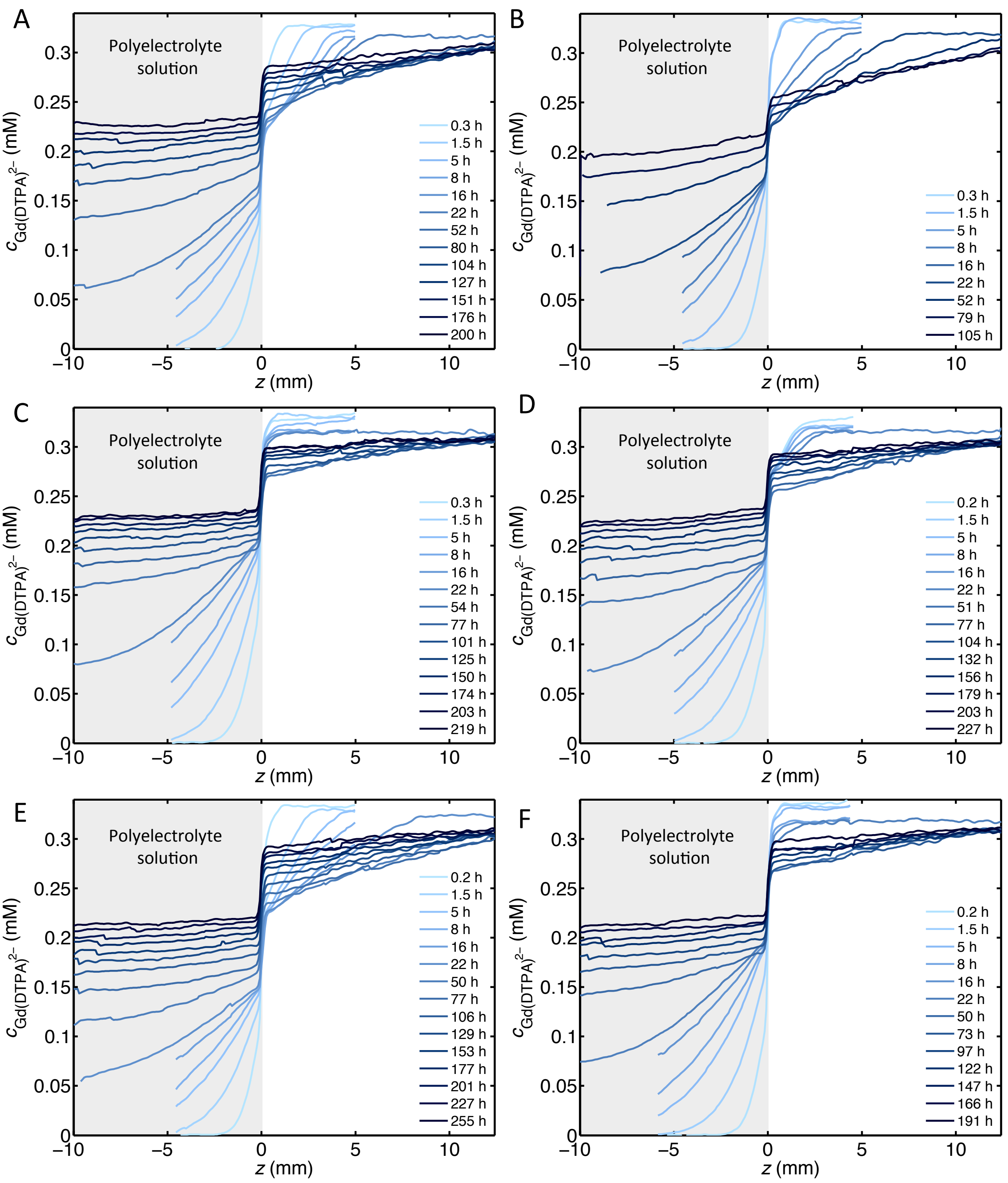

FIG. S1: Concentration profiles of $Gd(DTPA)^{2-}$ at different times in the salt solution and the polyelectrolyte solution ($z < 0$) with **(A)** - **(B)** FCD = -73 mM, **(C)** - **(D)** FCD = -92 mM and **(E)** - **(F)** FCD = -108 mM, $Gd(DTPA)^{2-}$ was injected in the salt solution at $t = 0$ and the data was acquired using μMRI.
