# Supplementary material for: Intermolecular interactions play a role in the distribution and transport of charged contrast agents in a cartilage model
Source: PLoS One. 2019 Oct 3;14(10):e0215047. doi: 10.1371/journal.pone.0215047 (PMC6776344; doi:10.1371/journal.pone.0215047)
Supplement: S2 Fig — (PDF) [file pone.0215047.s005.pdf]

S2 Figure. Additional concentration profiles from experiments and FEM simulations

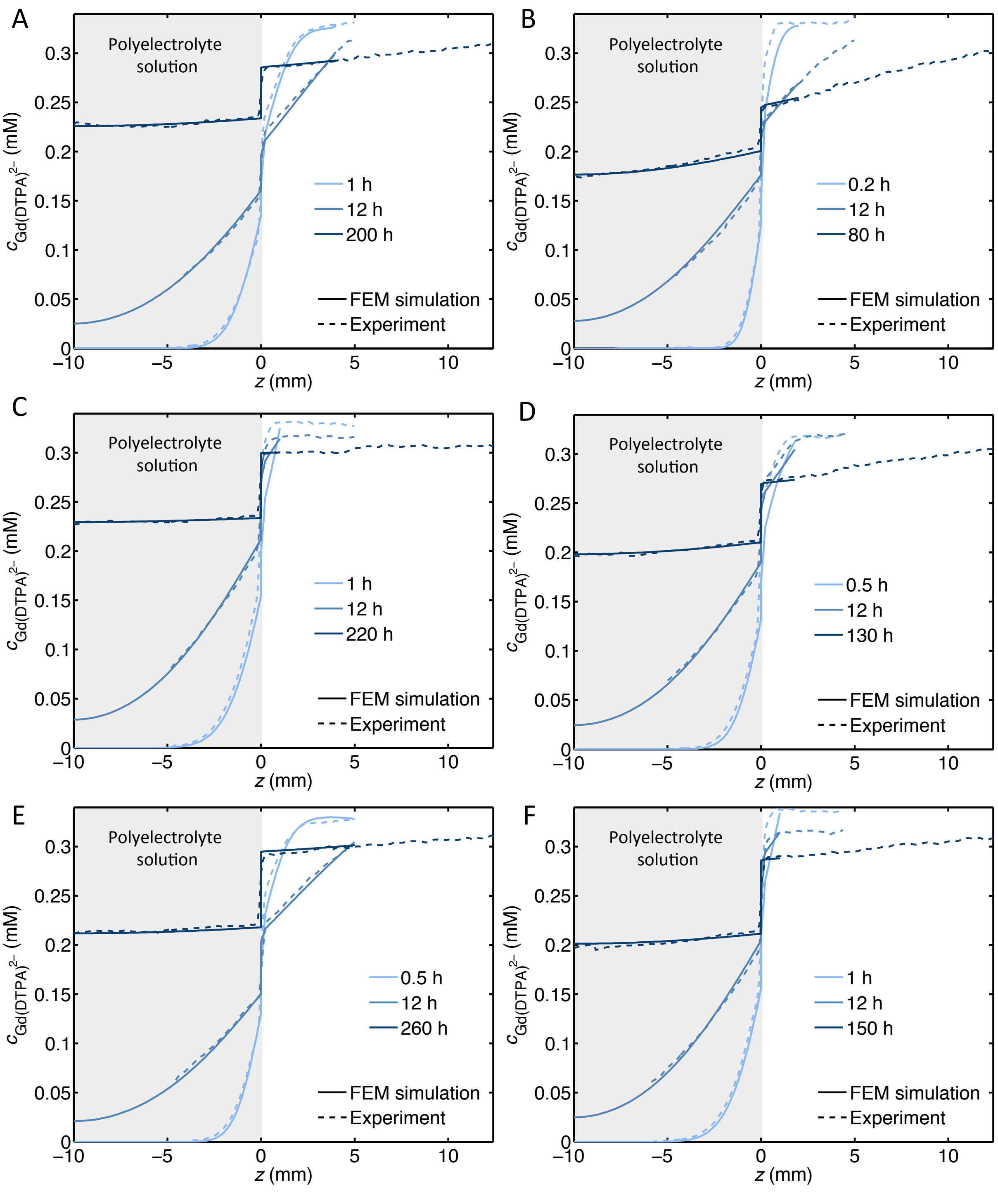

FIG. S2: Experimental (dashed) and simulated (solid) values for the concentration of $Gd(DTPA)^{2-}$ at different positions along the container, z, for a polyelectrolyte solution with **(A)** - **(B)** FCD = -73 mM, **(C)** - **(D)** FCD = -92 mM and **(E)** - **(F)** FCD = -108 mM.
