# Supplementary material for: Intermolecular interactions play a role in the distribution and transport of charged contrast agents in a cartilage model
Source: PLoS One. 2019 Oct 3;14(10):e0215047. doi: 10.1371/journal.pone.0215047 (PMC6776344; doi:10.1371/journal.pone.0215047)
Supplement: S3 Fig — (PDF) [file pone.0215047.s006.pdf]

S3 Figure. Obtained values of $\Delta\mu_i^{ex}$ from Monte Carlo simulations

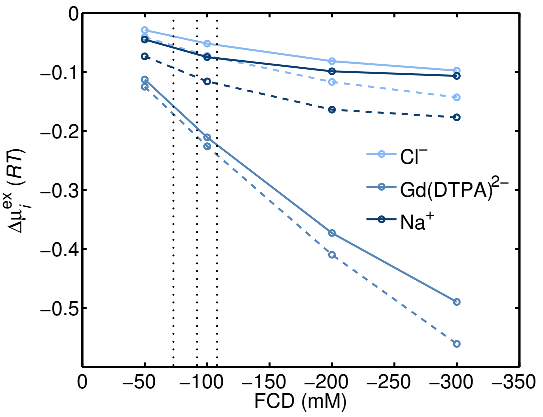

FIG. S3: Obtained values of $\Delta\mu_i^{ex}$ from Monte Carlo simulations plotted against FCD. Data for Na^+ and $Gd(DTPA)^{2-}$ is taken from ref. (Algotsson 2012). Solid lines are results with a value of 1.2 nm as the distance between the monomers representing the polyelectrolyte and dashed lines are results with a corresponding value of 0.8 nm. In this study the value for $\Delta\mu_{Na^+}^{ex}$ was obtained from the curve with dashed line. The vertical dashed lines is a guideline for the eye and represent the FCDs used in this study.
